# Advanced Glycation End Product-Induced Astrocytic Differentiation of Cultured Neurospheres through Inhibition of Notch-Hes1 Pathway-Mediated Neurogenesis

**DOI:** 10.3390/ijms15010159

**Published:** 2013-12-23

**Authors:** Yijing Guo, Pin Wang, Haixia Sun, Rongrong Cai, Wenqing Xia, Shaohua Wang

**Affiliations:** 1 Department of Neurology, Affiliated ZhongDa Hospital of Southeast University, No.87 DingJiaQiao Road, Nanjing 210009, China; E-Mail: Janegyj@aliyun.com; 2 Department of Endocrinology, Affiliated ZhongDa Hospital of Southeast University, No.87 DingJiaQiao Road, Nanjing 210009, China; E-Mails: wangpin542@126.com (P.W.); sunshine_9092@163.com (H.S.); rongrong19900710@163.com (R.C.); wen_qing_xia@126.com (W.X.)

**Keywords:** advanced glycation end products, neural stem cells, differentiation, Notch-Hes1 pathway

## Abstract

This study aims to investigate the roles of the Notch-Hes1 pathway in the advanced glycation end product (AGE)-mediated differentiation of neural stem cells (NSCs). We prepared pLentiLox3.7 lentiviral vectors that express short hairpin RNA (shRNA) against Notch1 and transfected it into NSCs. Cell differentiation was analyzed under confocal laser-scanning microscopy. The percentage of neurons and astrocytes was quantified by normalizing the total number of TUJ1^+^ (Neuron-specific class III β-tubulin) and GFAP^+^ (Glial fibrillary acidic protein) cells to the total number of Hoechst 33342-labeled cell nuclei. The protein and gene expression of Notch-Hes1 pathway components was examined via western blot analysis and real-time PCR. After 1 week of incubation, we found that AGE-bovine serum albumin (BSA) (400 μg/mL) induced the astrocytic differentiation of cultured neurospheres and inhibited neuronal formation. The expression of Notch-Hes1 pathway components was upregulated in the cells in the AGE-BSA culture medium. Immunoblot analysis indicated that shRNA silencing of Notch1 expression in NSCs significantly increases neurogenesis and suppresses astrocytic differentiation in NSCs incubated with AGE-BSA. AGEs promote the astrocytic differentiation of cultured neurospheres by inhibiting neurogenesis through the Notch-Hes1 pathway, providing a potential therapeutic target for hyperglycemia-related cognitive deficits.

## 1. Introduction

Type 2 diabetes mellitus (DM) is common among the elderly and it has been associated with cognitive deficits and dementia [1–4]. DM increases the risk of Alzheimer’s disease (AD) through several biologically plausible pathways, such as hyperglycemia. Hyperglycemia disrupts redox reaction that leads to neuronal damage in the central nervous system, which may contribute to behavioral impairments and memory disturbances [5,6].

Endogenous advanced glycation end products (AGEs) are produced by the Maillard reaction, wherein the carbonyl group of carbohydrates reacts non-enzymatically with the primary amino groups of proteins [7–9]. Toxic AGEs quickly accumulate during hyperglycemia, during oxidative stress, which contributes to the pathophysiology of aging [10], and as a complication of diabetes [11–13]. AGEs accumulate in the nerves of diabetics, and inhibiting the formation of AGEs using anti-glycation agents improves neuropathic changes in diabetic rats [14]. The studies found that AGEs played roles in several possible mechanisms that link diabetes to AD and vascular dementia, including accelerated neuronal damage [15–17] and vascular injury [16,18]. Our recent study found that AGE-bovine serum albumin (BSA) downregulates the proliferation and neurogenic differentiation of neural stem cells (NSCs) in a dose-dependent manner [19]. Furthermore, our unpublished data show that incubating NSCs with AGE-BSA promotes astrocytic formation.

NSCs are capable of forming multipotent neurospheres when cultured *in vitro*. Neurospheres are self-renewing and they differentiate to form specific neurons, glial cells, and oligodendrocytes [20,21]. Intriguingly, the adult hippocampus retains the capability to produce new neurons throughout life [22]. Moreover, diabetes impairs hippocampal neurogenesis in insulin-deficient rats, insulin-resistant mice [23], and in streptozotocin-treated diabetic rats [24]. These findings raise the possibility that AGEs impair endogenous neural regeneration, thereby causing the cognitive deficits in diabetes. The detailed mechanisms are still under investigation.

The Notch1 pathway is involved in hippocampal neurogenesis, both under physiologic [25] and pathologic conditions [26]. Mammals have *Notch1* to *Notch4* genes and five *Delta*, *Serrate*, *Lag2* (DSL) ligand genes (*Dll1*, *Dll3*, *Dll4*, *Jag1*, and *Jag2*) [27]. Ligand binding induces proteolytic cleavage of the Notch receptor, releasing the *Notch* intracellular domain (NICD) to translocate into the nucleus. NICD directly modulates transcription factor function and a series of downstream target genes, including the *Hes1* gene [28,29]. This pathway is required for maintaining and expanding the NSCs pool [30] and also regulates NSC differentiation by inhibiting their neuronal fate while subsequently promoting glial fate [31,32].

In this study, we prepared pLentiLox3.7 lentiviral vectors that express *short hairpin RNA* (shRNA) against *Notch1* and transduced NSCs to determine whether AGEs affect NSC differentiation by activating the *Notch* signaling cascade. Cell differentiation was analyzed under a confocal laser-scanning microscope. The protein and gene expression of Notch-Hes1 pathway components were examined via western blot analysis and real-time PCR (RT-PCR). We tried to determine the roles of the Notch-Hes1 signaling cascade to block the effects of AGEs on neuronal differentiation. Thus, this pathway may be a therapeutic target for hyperglycemia-related cognitive deficits.

## 2. Results and Discussion

### 2.1. Reduced Efficacy of shRNAs

**Figure 1.**
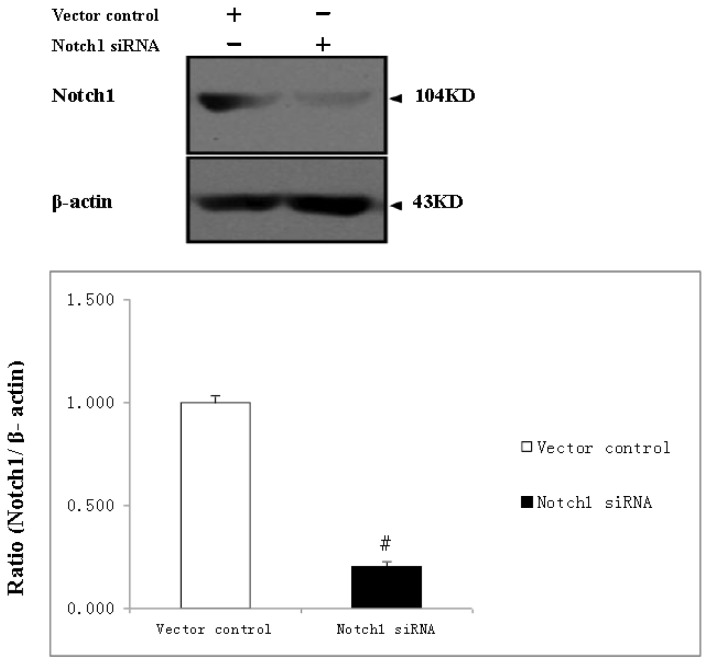
Knock-down efficiency of the shRNAs. Notch1 protein level by Western blotting indicates that shRNAs delivered by lentiviral vectors silenced *Notch-1* expression in NSCs, β-actin staining was used as a control for equal protein loading. # indicates *p <* 0.01, Notch-1 siRNA NSCs *vs.* Vector control.

As confirmed by RT-PCR, *Notch1-sh2RNA* delivered by lentiviral vectors effectively silenced *Notch1* expression in PC12 cells. Thus, the vector containing *Notch1-sh2* can be used for further research. shRNA delivered into NSCs via lentiviral vectors were used to functionally silence *Notch1* expression, as confirmed by western blot analyses. Immunoblot analysis indicated that the protein level of *Notch1* decreased by 80.7% when NSCs were infected with *Notch-1* shRNA virus (Figure 1).

### 2.2. Effect of Notch-Hes1 Pathway Interference on the Differentiation of NSCs Incubated with AGE-BSA

**Figure 2.**
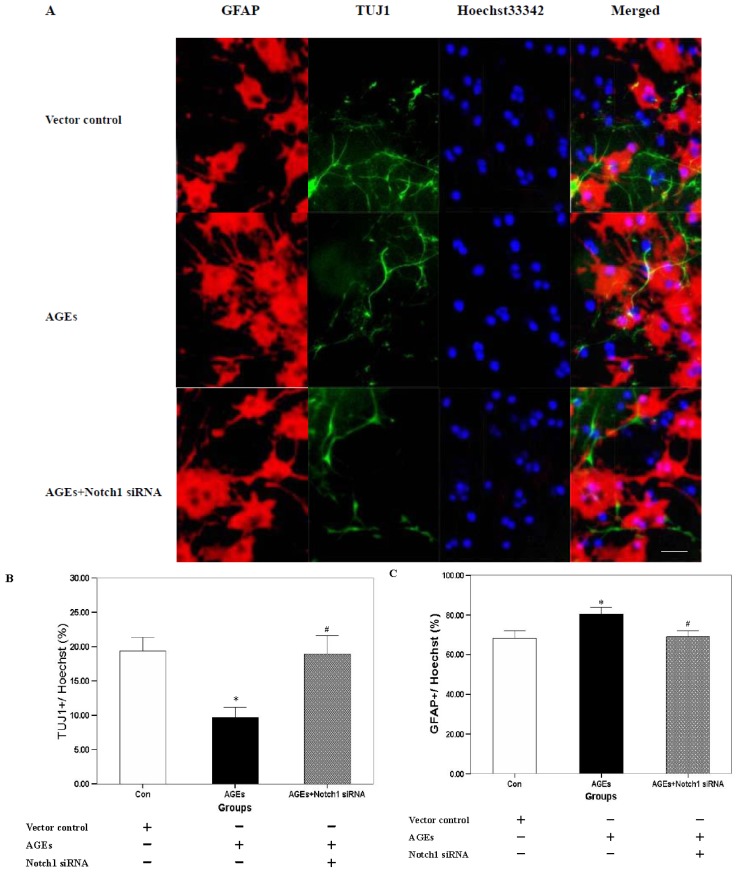
AGEs stimulated the differentiation of cultured neurospheres into astrocytes by inhibiting neuronal differention through Notch-Hes1 pathway. (**A**) NSCs derived from SGZ could generate into neurons (TUJ1) and astrocytes (GFAP) after seven days of culture in differentiation medium. Scale bar, 50 μm; and (**B**,**C**) 400 μg/mL AGE-BSA induced a significant decrease in the proportion of TUJ1 cells and increase in the numbers of GFAP-immunoreactive cells with astrocytic morphology (* *p* < 0.05, *n* = 3). All these values were reversed after *Notch1*-silenced NSCs were incubated with 400 μg/mL AGE-BSA (^#^ *p* < 0.05, *n* = 3).

Dissociated NSCs were differentiated for a week to determine their ability to generate multiple neural cell lineages. As shown in Figure 2, AGE-BSA (400 μg/mL) suppressed the numbers of TUJ1-immunoreactive cells with neuronal morphology (9.7% ± 1.5% *vs.* 19.3% ± 2.1%, *n* = 3, *p* < 0.01) and promoted the number of GFAP-immunoreactive cells with astrocytic morphology (80.7% ± 3.2% *vs.* 68.3% ± 3.5%, *n* = 3, *p* < 0.05). All these values reversed after the Notch1-silenced NSCs were incubated with 400 μg/mL AGE-BSA (19.0% ± 2.6% *vs.* 9.7% ± 1.5%, 69.0% ± 3.0% *vs.* 80.7% ± 3.2%, *n* = 3, *p* < 0.01 and *p* < 0.05, respectively).

### 2.3. Expression of Notch-Hes1 Components of the NSCs Incubated with AGE-BSA

**Figure 3.**
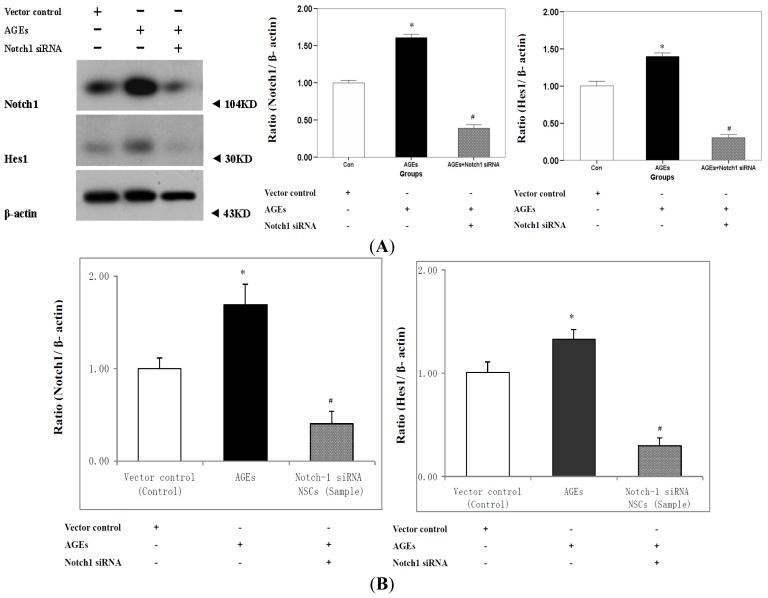
AGEs stimulated the protein and gene expression of Notch-Hes1 pathway in cultured neurospheres. (**A**) Western blotting shows that AGE-BSA increased protein expression of NICD and Hes1 in the NSCs (Vector control) after 7 days of culture in differentiation medium (* *p* < 0.05, *n* = 3). AGE-BSA caused a significant decrease in the protein expression of NICD and Hes1 in Notch1-silenced NSCs compared with that in NSCs (^#^ *p* < 0.05, *n* = 3); and (**B**) Real-time PCR shows that AGE-BSA increased gene expression of NICD and Hes1 in the NSCs (Vector control) after 7 days of culture in differentiation medium (* *p* < 0.05, *n* = 3). AGE-BSA caused a significant decrease in the gene expression of NICD and Hes1 in Notch1-silenced NSCs compared with that in NSCs (^#^ *p* < 0.05, *n* = 3). Protein level was examined by Western blotting, β-actin staining was used as a control for equal protein loading. Gene level was examined by real-time PCR normalized to β-actin.

As shown in Figure 3, AGE-BSA increased protein expression of Notch1 in the NSCs (vector control). We then assessed the downstream target Hes1, and its protein expression was upregulated in AGE-BSA culture medium. The Notch1 protein expression was upregulated 16.4% and the Hes1 expression was upregulated 27.7% (all *p* < 0.001). *Notch1* gene expression decreased 25.9% and *Hes1* gene expression 46.2% (all *p* < 0.001) relative to vector control.

AGE-BSA decreased the protein and gene expression of Notch1 and Hes1 in Notch1-silenced NSCs compared with normal NSCs. Notch1 protein expression was downregulated 19.6% and Hes1 protein expression was downregulated 27.8% (all *p* < 0.001), whereas *Notch1* gene expression decreased by 28.4% and *Hes1* gene expression decreased by 77.4% (*p* < 0.001).

### 2.4. Discussion

Our results demonstrate that AGEs favor the differentiation of cultured neurospheres into astrocytes and inhibit neuron formation. The Notch-Hes1 signaling pathway plays an important role in mediating the aforementioned AGE-related differentiation. To confirm the effect of Notch1 deficiency, lentiviral vector-based RNAi was prepared to knock down Notch1 expression in NSCs. RNA interference using shRNA is probably the most common gene-silencing strategy for post-transcriptional regulation.

In accordance with our recent findings [19], our present study demonstrates that AGEs suppress neurogenic differentiation of NSCs *in vitro.* The hippocampus retains robust neurogenesis that generates several thousand new neurons daily in the dentate gyrus during the adulthood [33]. These new neurons develop morphologic and functional properties of dentate granule cells and are functionally recruited into the dentate gyrus circuitry to form appropriate synapses with already existing neurons [33–35].

Neurogenesis in the adult dentate gyrus occurs in response to diverse physiologic stimuli [36] and brain injury, including stroke [37] and seizures. Although the function of hippocampal neurogenesis in adults remains unknown, it may be a critical element in brain repair. Additionally, inhibition of neurogenesis in the hippocampus may be linked to the cognitive deficits of aging [38] and AD [39]. These findings suggest that new neurons [40] play an important role in behavioral plasticity, including learning process [40]. Taken together, inhibited neuron formation in response to AGEs suggests that hyperglycemia-impaired neurogenesis occurs in the adult dentate gyrus and probably contributes to diabetes-related cognitive deficits.

The approximately 10% decrease in the number of neurons induced AGE-BSA coincides with the 10% increase in the number of glial fibrillary acidic protein (GFAP)-immunoreactive cells with astrocytic morphology. This result indicates that AGE-BSA induces the differentiation of NSCs in to astrocytes. Astrocytes are the most abundant cell type in the central nervous system, constituting about 20% to 50% of the human brain volume, depending on the brain region. They provide metabolic and trophic support to neural cells, modulate synaptic activity, and protect neurons against injury [41]. Several studies have shown that diabetes affects astrocytes [42,43].

Astrocytes may exhibit the earliest and potent cellular reaction in various damaging factors; hence, AGE-induced NSC differentiation into astrocytes occurs in response to hyperglycemia. Unlike most other organs in the body, the brain usually does not respond to injury by forming a fibrous scar (formed by fibroblasts through collagen deposition). Instead, the brain forms a glial scar consisting of reactive astrocytes. The presence of reactive astrocytes is often referred to as astrogliosis or just gliosis. A key indicator of glial reactivity is increased synthesis of GFAP, an intermediate filament protein of the astrocytic cytoskeleton. Chronic reactive gliosis exacerbates diabetic neuropathy in uncontrolled hyperglycemia during diabetes [44,45]. GFAP levels have been correlated with excessive ROS generation [42]. The results of the current study confirm that increased GFAP levels are indicative of astrocyte reactivity caused by AGE-induced oxidative stress and a sensitive biomarker for neurotoxicity assessment.

We also found that AGE-BSA stimulates the gene and protein expression of components of the Notch-Hes1 pathway; activation of this pathway seems to explain how AGE-BSA induces the differentiation of NSCs in to astrocytes. RNAi knock down of Notch1 expression in NSCs reversed the effects of AGE-BSA on differentiation, which indicates that the Notch-Hes1 pathway is involved in AGE-induced differentiation of cultured neurospheres into astrocytes by inhibiting new neuron formation.

This study has certain limitations in the data interpretation. First, multipotent NSCs differentiate into neurons, astrocytes, and oligodendrocytes under certain culture conditions. However, the effects of AGEs on oligodendrocytes from NSCs were not evaluated under the tested conditions. In addition, only one time point and one AGE-BSA concentration were tested in the current study. Despite these limitations, the findings raise the possibility that AGEs dramatically affect the fate of NSCs by stimulating glial lineage selection and inhibiting the neuronal lineage, potentially through the Notch-Hes1 pathway. Reactive astrogliosis is a cellular response to brain injury, such as hyperglycemia. This pathway is thus a potential therapeutic target for hyperglycemia-related cognitive deficits. Further investigations are required to determine the efficacy of this approach as a potential neuroprotective strategy based on our findings.

## 3. Experimental Section

### 3.1. Preparation of a Lentiviral Vector Expressing shRNA against Notch1

Two shRNAs were designed based on the nucleotide sequence of the *Notch1* gene using the online tool provided by Ambion (http://www.ambion.com, Austin, TX, USA). The target sequences for siNotch1 are as follows: sh1: 5′-GCAACCTTCAGTGTAATAA-3′; sh2: 5′-GAGGAAGACAAGCATTACT-3′. The nonspecific scrambled sequence used as the control was 5′-CCUACGCCAAUUUCGU-3′. Annealed oligonucleotides (1 μL), 2 pmol of *Hpa*I/*Xho*I digested vector pLL3.7, 1 μL of 10× ligation buffer, 1 μL of T4 DNA ligase (TaKaRa, Dalian, China), and ddH_2_O were mixed together and incubated overnight at 4 °C. A total of 5 μL of the ligation products were transfected into competent *E. coli* DH5α (TaKaRa). pLL3.7 or pLL3.7/shRNA (10 μg) and 10 μg of packing mix pSPAX2 and pMD2.G (Invitrogen, Austin, TX, USA) were mixed together and transfected into the 293T cells to produce replication-defective lentiviruses. The culture supernatants were collected 48 h after transfection and filtered through a 0.45 μm-syringe filter and stored at −80 °C.

### 3.2. Cell Culture and Transfection

NSCs were prepared as described [19]. Primary proliferative neurospheres were formed and then passaged by mechanical dissociation. The dissociated NSCs were subcultured at 5 × 10^4^ cells/mL in a culture bottle to synchronize the cells for transfection. Several days after NSC transfection, the NSCs formed neurospheres in the uncoated culture bottles in the NSC culture medium, which indicated their undifferentiated state. The culture medium consisted of Dulbecco’s modified Eagle’s/F-12 (1:1) medium (Gibco, Austin, TX, USA) containing 0.6% glucose, 0.1% NaHCO3, 5 mM HEPES (Invitrogen, Austin, TX, USA), 10 mg/mL N2 supplement (Gibco, Austin, TX, USA), 20 mg/mL B27 supplement (Gibco, Austin, TX, USA), 20 ng/mL human recombinant epidermal growth factor (Promega, Madison, WI, USA), and 20 ng/mL human recombinant basic fibroblast growth factor (Promega, Madison, WI, USA).

### 3.3. AGE-BSA Preparations

AGE-BSA was prepared by incubating 5.0 g/L BSA (Amresco, Cleveland, OH, USA) with 0.5 mol/L glucose (Sigma, Oakville, ON, Canada) at 37 °C for 90 days under sterile conditions and finally dialyzed against PBS for 48 h, as previously described [46,47]. Control non-glycated BSA was incubated under the same conditions without glucose. AGE-specific fluorescence determinations were performed by measuring light emission at 440 nm with excitation at 370 nm using a fluorescence spectrophotometer (F-3000, Hitachi, Japan). AGE-BSA was added into the aforementioned culture medium containing 10% fetal bovine serum (Gibco, Austin, TX, USA) for the differentiation study. Vector control and Notch1 RNAi-transfected NSCs were synchronized and incubated for 7 d in AGE-BSA (400 μg/mL) before the NSCs were evaluated for differentiation and the Notch-Hes1 pathway was measured. Vector control NSCs treated with non-glycated BSA were designated as the control.

### 3.4. Evaluation of Differentiation

Dissociated NSCs from neurospheres were seeded on 200 μg/mL poly-l-lysine-coated coverslips at 1 × 10^4^ cells/mL in DMEM/F12 containing 10% fetal bovine serum. After 7 days, the cells were fixed with 4% paraformaldehyde in phosphate-buffered saline (PBS) for 10 min, permeabilized in PBS containing 0.1% Triton X-100 for 30 min, and then rinsed with PBS. The fixed cells were incubated with antibodies against neuron-specific β-3 tubulin (TUJ1; 1:500, Chemicon, Billerica, MA, USA) and GFAP (1:1000, Invitrogen, Austin, TX, USA). Percentage of TUJ1^+^ and GFAP^+^ cells were quantified by normalizing the total number of TUJ1^+^ and GFAP^+^ cells to the total number of cell nuclei labeled with Hoechst 33342 (Sigma, Oakville, ON, Canada). The process was repeated three times, and at least 8 to 10 coverslips (each group) were examined each time.

### 3.5. Protein and Gene Expression of Notch-Hes1 Pathway Components

After 7 days of differentiation, the protein expression of Notch-Hes1 pathway components was examined using western blot analysis, Notch1 on 8% SDS polyacrylamide gels, and Hes1 on 12% SDS polyacrylamide gels. Proteins were then transferred onto polyvinylidene fluoride membranes according to the manufacturer’s instructions (Invitrogen, Austin, TX, USA), and immunoblotted using the following antibodies: Notch1-ICD (rabbit anti-rat polyclonal IgG, 1:1000; Abcam, Cambridge, UK), Hes1 (rabbit anti-rat polyclonal IgG 1:1000, Santa Cruz Biotechnology, Dallas, TX, USA), and β-actin (rabbit anti-rat polyclonal IgG 1:2000; Santa Cruz Biotechnology, Dallas, TX, USA). The secondary antibody was goat anti-rabbit IgG (1:5000, Abcam, Cambridge, UK).

Real-time RT-PCR was performed by monitoring the increase in fluorescence of SYBR Green dye (Invitrogen, Austin, TX, USA) using a Rotor-Gene 3000 real-time PCR apparatus (Corbett Research, New South Wales, Australia) according to the manufacturer’s instructions. All PCR primers were designed using Primer Express Software V2.0 (Roche Molecular System, Pleasanton, CA, USA, 2001) (Table 1). All measurements were conducted in triplicate. The results of the real-time RT-PCR are expressed as *C*t values, where *C*t is defined as the threshold cycle of PCR at which the amplified product was first detected. To minimize intra-assay and inter-assay variability caused by differences in PCR efficiency, the quantity of the target gene was normalized to the amount of β-actin cDNA. The *C*t or threshold value of the target sequence is directly proportional to the absolute concentration compared with the threshold value for reference genes. The relative mRNA expression level of the target gene was plotted as fold change compared with the control, as determined using the 2^−ΔΔ^*^C^*^t^ method [48], a relative quantification algorithm. The factor *X* by which the amount of the changed gene can be calculated with the following formula:

(1) $$X=2^{-\Delta \Delta C\text{t}}$$

where ΔΔ*C*t = (*C*t_target gene_ − *C*t_β-actin_) control − (*C*t_target gene_ − *C*t_β-actin_) sample.

### 3.6. Statistical Analysis

The data were analyzed using Graph Pad Prism version 4.0 (GraphPad Software, San Diego, CA, USA, 2003). Values are presented as means ± SD. A Student’s *t* test or Bonferroni correction was performed for statistical evaluation. Differences with *p* < 0.05 were considered statistically significant.

## 4. Conclusions

AGEs affect the fate of NSCs by stimulating glial lineage selection and inhibiting the neuronal lineage, potentially through the Notch-Hes1 pathway. That is, AGEs promote the astrocytic differentiation of cultured neurospheres by inhibiting neurogenesis the through Notch-Hes1 pathway, providing a potential therapeutic target for hyperglycemia-related cognitive deficits.

## Figures and Tables

**Table 1.** Sequence for quantitative real-time PCR primers.

Genes	Primers
*Notch-1-F*	5′-CCGCTGTGAGTCGGTCATTA-3′
*Notch-1-R*	5′-GGCACCTACAGATGAATCCA-3′
*Hes1-F*	5′-TTCAGCGAGTGCATGAACGA-3′
*Hes1-R*	5′-GTAGGTCATGGCGTTGATCT-3′
*β-actin-F*	5′-CCTAGGCACCAGGGTGTGAT-3′
*β-actin-R*	5′-TTGGTGACAATGCCGTGTTC-3′
